# The Curious Case of ZEB1

**DOI:** 10.15190/d.2018.7

**Published:** 2018-12-31

**Authors:** Mecca Madany, Tom Thomas, Lincoln A. Edwards

**Affiliations:** Department of Neurosurgery, Cedars-Sinai Medical Center, Los Angeles, CA, USA; Department of Pathology, Brigham & Women’s Hospital, Harvard Medical School Boston, MA, USA; Feil Family Brain and Mind Research Institute, Weill Cornell Medicine, New York, NY, USA

**Keywords:** ZEB1, glioma stem cells, glioblastoma.

## Abstract

The Zinc Finger E-box binding homeobox (ZEB1/TCF8 or DeltaEF1) is at the forefront of transcription factors involved in controlling epithelial-to-mesenchymal transitions (EMT). Essentially, EMT allows for the reorganization of epithelial cells to become migratory cells with a mesenchymal phenotype.  In addition to ZEB1 being involved in embryonic development, ZEB1 has also been linked to processes involving micro-RNAs, long non-coding RNAs and stem cells. In recent years there has been an accumulation of evidence with regard to ZEB1 in various cancers. Although increased ZEB1 expression has largely been associated with EMT, cancer invasion, and tumorigenicity, there have been some episodic reports that have gone against the traditional reporting of the role of ZEB1. Indicating that the function of ZEB1 and the mechanisms by which ZEB1 facilitates its activities is more complex than was once appreciated. This complexity is further exacerbated by the notion that ZEB1 can act not only as a transcriptional repressor but a transcriptional activator as well. This review seeks to shed light on the complexity of ZEB1 with respect to cancer.

## **1. Introduction**

The EMT process is a critical component in the developmental stages that include but are not limited to gastrulation, heart morphogenesis, and neural crest formation. EMTs do not just revolve around early developmental processes however, but also encompass adult tissues involving wound healing, fibrosis, and cancer^1-4^. The EMT process is activated by a host of genes including, TGF-β, TWIST, SNAIL, HGF, and ZEB1^5-7^. EMT is associated with the normal functions that normal cells undergo; however, tumor cells can also utilize the EMT process for the purposes of gaining metastatic activity. The upregulation of the transcription factor ZEB1 in particular in the EMT process has coincided with metastasis. In contrast, metastasis can be attenuated with inhibition of ZEB1 activity which is not true for other EMT genes such as SNAIL and TWIST^8,9^. ZEB1 has been implicated in neoplastic transformation, tumor progression, and metastasis in several different tissue types including lung, breast, and colon cancer. Both lung cancer and breast cancer have been shown to have high levels of ZEB1 expression and are associated with metastasis and poor patient outcome^10^. Mechanistically, ZEB1 has been shown to bind promoters of epithelial polarity genes including Crumb3, HGL2, and PATJ (Pals1-associated tight junction), resulting in their repression and the invasive shift in breast cancer cells^11^. Similar functions have been seen in colorectal cancer where ZEB1 promotes a metastatic shift and loss of cellular polarity by repressing the expression of the polarity factor Lgl2^12^. It is currently hypothesized whether tumor cells have some dependency on early EMT developmental genes during metastatic activation^13^. It would appear that ZEB1 gives evidence to such a theory, as ZEB1 is not only associated with embryogenesis but metastatic activation also. In addition, there is a great deal of interplay between EMT associated genes in the EMT process. For example, TGF-β and the TGF-β pathway are important for the activation of ZEB1^14^. Yet ZEB1 is also tightly controlled by micro-RNAs such as miR-200^15,16^ (along with miR-200 family members miR-200a, miR-200c and miR-141), which are involved in a negative feedback loop to control EMT during normal development and tumorigenesis^17^. The complexity of ZEB1 goes deeper than the interplay with growth factors, micro-RNAs, embryogenesis, and EMT activation but also goes to the function of ZEB1 itself, which can act as both a transcriptional repressor and a transcriptional activator. Activation appears to occur with coactivators such as p300 and P/CAF whereas repression appears to occur with corepressors such as CtBP^18^. In addition, ZEB proteins (the two in vertebrates are ZEB1 and ZEB2) ZEB1 and ZEB2 can act antagonistically to each other; such is the case in TGF-β/BMP signaling^19^. ZEB1 even under normal conditions can be very complex with opposing functional activities so it is not unreasonable to believe that in cancer the complexity of ZEB1 would remain, and in fact be greater due to ZEB1 dysregulation. In this review we address the complexity and discrepancies with respect to the functional roles of ZEB1 in cancer, with a focus on brain cancer of both low and high-grade gliomas.

## **2. ZEB1 Activation and Repression**

A multifunctional transcription factor, ZEB1 contains zinc finger domains, a central homeodomain as well as multiple protein binding domains, which allow for binding of both DNA and proteins. The two-end terminal zinc finger clusters are a defining characteristic of ZEB1 that allows it to bind to E-box and Z-box DNA sequences. ZEB1’s protein domains include the CtBP and SMAD interaction domains (CID and SID respectively)^18^ as well as p-300/P-CAF domain. Through these domains ZEB1 is able to engage co-activators and co-repressors leading to the up or downregulation of target genes^18,19^. As multiple studies have shown, ZEB1 may play a dual role given its ability to be transcriptionally repressive and activating.

The first identification of ZEB1 in chick embryos found it to be a repressor of d1-crystallin enhancer and suggested that it played a role in the development of embryos^20^. This role was confirmed in studies with mice that indicated ZEB1 null mice died perinatally marked by skeletal defects and a severe T cell deficiency^21^. To date the most well studied suppression activity of ZEB1 is its ability to suppress the CDH1 gene, which encodes for E-Cadherin, by binding to the E-box found in the CDH1 promoter region^22,23^. This binding leads to the recruitment of CtBP corepressors ultimately resulting in repressed CDH1 transcription and leading to epithelial mesenchymal transition (EMT), a hallmark of tumor invasion and metastasis. It has also been revealed that the repression of E-Cadherin is possible without CtBP as ZEB1 is able to use BRG1 as a replacement corepressor^24^. ZEB1 is able to activate the transcription of TGF-β-responsive genes via its SID domain by recruiting SMAD and p300 proteins^25^.

ZEB1 targets and regulates a large list of genes in a variety of cell types although; the specific mechanisms of action remain largely unknown. Multiple binding sites for co-repressors and co-activators that are also able to produce post-translational modifications suggest a variety of intricate means of action. A study done in MDA MB-231 breast cancer cells^26^ revealed that knocking down ZEB1 produced downregulation of more than 30 genes and upregulation of more than 200, many of which affected epithelial cell adhesion genes including the classic cadherin superfamily, components of tight junctions (e.g. occluding, claudin 7) desmosomes such as desmoplakin, plakohilin3 and desmocollin 2 and gap junctions such as connexin 26 and 31. Genes also affected by ZEB1 knockdown involved cellular differentiation^26^. ZEB1 regulates p73 in mesenchymal cells by repressing it and is also able to repress the expression of interleukin 2 and CD4 in hematopoietic cells^27,28^. ZEB1 represses collagen in osteoblasts and has also been shown to inhibit muscle differentiation by blocking the transcriptional activity of the myogenic factor MEF2C^29^. ZEB1’s ability to be a transcriptional repressor or a transcriptional activator goes beyond what has been seen in normal regulatory processes, being also found within dysregulated processes such as cancer.

## **3. ZEB1 Activation and Repression in Cancer**

Within the context of cancer, irregular expression of ZEB1 has been found in a multitude of human cancers including breast, pancreatic, colon, gastric, lung, uterine, liver, lymphoma, and brain cancers^30-35^. ZEB1 has been shown to upregulate or downregulate genes that play roles in cancer progression, invasion, migration, and metastasis. These result in a cumulative effect of ZEB1 on patient survival. Indeed, in a study on pancreatic ductal adenocarcinoma it was found that high expression of ZEB1 in the cancer cells as well as cancer associated fibroblasts inversely correlated to patient prognosis^31^. Similar results of overexpression were found in colorectal cancer and uterine cancer^30^. More in depth studies on ZEB1 expression and the development of gastric cancer found that in addition to affecting invasion and metastasis the overexpression of ZEB1 may actually be related to its development and occurrence when comparing gastric carcinoma to normal gastric mucosa tissues^34,36^.

It has been well established that E-Cadherin functions as a tumor suppressor gene that, when lost, causes cancer cells to increase metastasis, migration and invasion in addition, migration, invasion, and metastasis negatively impact patient survival^37^. As mentioned, this loss of E-Cadherin is a hallmark characteristic of EMT. Given the significant control that ZEB1 has in repressing E-Cadherin, it therefore serves as a driver of EMT and cancer progression. In vivo studies^38^ have shown that tumors with increased ZEB1 and lowered E-Cadherin were associated with advanced disease and metastasis as well as poorer survival with many studies on the subject cementing the role of ZEB1 in EMT and tumor progression. This role of ZEB1 has been further confirmed using cancer cell lines. Within non-small cell lung cancer (NSCLC) cells it was found that with the suppression of E-Cadherin, ZEB1 has the ability to increase invasion via transcriptional activation of MMP-2 as well as cause aberrant EGFR signaling. In addition to cell lines, NSCLC tissues were found to have a loss of E-Cadherin and activated ERK signaling (which leads to increased ZEB1 signaling) at the invasive tumor front^39^.

In addition to E-Cadherin, ZEB1 exhibits transcriptional control over other target genes that play roles in cancer progression. In breast and colorectal cancer ZEB1 is able to impact cell-cell adhesion and epithelial differentiation via key genes including the cell polarity genes HUGL2, Crumbs3 and PATJ (Pals1-associated tight junction) by binding to their promoters and repressing their transcription^40^. This repression is associated with primary tumor progression towards metastasis. Likewise, ZEB1 causes loss of cell polarity and increased metastasis in colorectal cancer by suppressing polarity genes, in particular Lgl2 (lethal giant larvae homolog 2), which leads to reduced cell-cell adhesion and increased invasion in cancer cell lines^40^. A study in Mantle Cell lymphoma (MCL) indicated that ZEB1 knockdown in MCL cells resulted in decreased proliferation in vitro and tumor growth in xenograft mouse models^41^. It shows that ZEB1 can activate anti-apoptotic genes such as BCL2 and MCL1 as well as proliferative genes including MYC, MKI67, and CCND1. At the same time ZEB1 was able to inhibit pro apoptotic genes such as TP53 and BAX resulting in an overall increased resistance to chemotherapy.

A further area of study that has become significant is the role that ZEB1 plays in the transcriptional activities of microRNAs that play a role in tumorigenicity. As increasing evidence is found for both the tumor suppressive and tumor promoter roles of microRNAs in cancer, there has been a push for further delineation of the role of ZEB1 and the miR-200 family. This family of microRNAs has received much of the focus in regard to ZEB1 as studies in various cancer lines have shown that this family is the most upregulated when ZEB1 is knocked down. ZEB1 was shown to have a strong transcriptional inhibition of miR200c and miR141 in pancreatic, breast, and colorectal cancers; thereby reducing their tumor suppressive properties^42^. These results revealed that ZEB1 is involved in a feed-forward loop with the miR200 family that triggers EMT and cancer cell invasion. Alternatively, the loop might switch and induce the opposite effect, which may help to explain intra-tumoral heterogeneity.

As the majority of studies involving ZEB1 and its role in cancer showed poorer patient prognosis as well as increased migration and invasion it was groundbreaking when a leukemia study presented evidence that ZEB1 functioned as a tumor suppressor in adult T-cell leukemia/lymphoma (ATLL)^43^. In an attempt to delineate the molecular mechanism underlying leukemogenesis after viral infection, genetic and expression analyses revealed that the TCF8 gene (ZEB1) was frequently altered by epigenetic dysregulation. ZEB1 mutant mice confirmed the importance of this alteration by frequently developing invasive CD4+ T-cell lymphomas; additional in vitro studies showed the loss of ZEB1 expression in ATLL cells resulted in an escape from growth inhibition by TGF-β. The authors concluded that these results confirmed a novel role for ZEB1 in ATLL as a tumor suppressor.

While at first glance these results may appear unexpected, further reflection on the duality of ZEB1’s nature as activator and suppressor would suggest that it is able to affect divergent functions in both normal and cancer cells. The ability of ZEB1 to function as a tumor suppressor or tumor promoter would not be a first within the cancer field. TGF-β signaling plays a versatile role within cancers and in many studies has been shown to drive tumor progression as well as tumor suppression^44^. Similar examples of duality can be seen with the transcription factors Mouse double minute 2 homolog (MDM2) and Retinoblastoma-Associated Protein 1 (E2F1)^45,46^.

Additional studies have reiterated the behavior of ZEB1 as a tumor suppressor. In a subpopulation of PC-3 prostate cancer cells, a study found that the knockdown of ZEB1 increased expression of laminin-332, a pro-migratory molecule. Consequently, these cells exhibited increased migration^47^. A study in lung cancer found a genetic context to the dual effects of ZEB1 with ZEB1 serving as an oncogene in KRAS-mutated lung cancer but as a tumor suppressor in EGFR-mutated lung cancer cells^48^. As a tumor suppressor, ZEB1 suppressed growth by increasing microRNA-200 targets to antagonize ERBB3, a driver of mutant EGFR-dependent cell growth.

## **4. ZEB1 and Gliomas**

A similar conflict of ZEB1 duality can be found in gliomas as seen in several recent studies. Given the aggressive nature of GBM many studies have approached the role of ZEB1 in GBM as that of an oncogene that is involved in the progression and severity of the disease with a negative effect on patient prognosis^49^.

Initial studies of ZEB1 in GBM linked it to similar oncogenic properties that were shown in the previously discussed cancers. Among the first studies, ZEB1 expression was shown to have a connection to tumor invasion, initiation as well as therapy resistance in glioblastoma^49^. Indeed, the study believed that these processes in glioblastoma were intertwined and dependent on ZEB1. A study by Kahlert et al^50^, used surgical specimens of pediatric and adult brain cancers to show that ZEB1 expression was significantly increased in more invasive tumors. In glioblastoma stem cells they found that targeting ZEB1 blocked the invasion of glioblastoma cells in a hypoxia setting and slowed them in normoxia suggesting a key role for ZEB1 in promoting invasion in the tumor core^50^. Another set of in vitro and in vivo experiments in several types of glioma cell lines, including U251 and A172, showed that ZEB1 was highly expressed across the lines and that when knocked down smaller tumors were formed. In human specimens it was found that with increasing grade of glioma there was higher expression of ZEB1 and ZEB2. As ZEB positive cells were more abundant in specimens from patients with recurring glioma, it suggested that ZEB plays a role in a more aggressive phenotype. These results are indicative of a positive correlation between ZEB levels and histopathological grade as well as invasiveness^51^.

Further studies have attempted to delineate specific ZEB1 functions at the molecular level as the genes it regulates in glioblastoma as a tumor promoter remain poorly understood. Prex1, a guanine nucleotide exchange factor, is predictive of shorter glioblastoma patient survival when its expression is increased. Prex1 is among the genes activated by ZEB1 in GBM and an in vivo study showed that it promotes invasiveness of GBM cells^52^. Another set of experiments showed that the repression of PPP3CC correlated with glioma progression and was often decreased in gliomas. This was significant as they further revealed that ZEB1 inversely correlated with PPP3CC expression and confirmed that ZEB1 was able to bind to its promoter to repress its expression^53^. The knockdown of ZEB1 produced the opposite effects. A similar study showed that the decrease of TET2 (ten-eleven translocation 2), implicated in tumor suppression in multiple cancers, is frequent in gliomas. This study also showed that ZEB1 is increased in gliomas and positively correlates with progression and inversely correlates with TET2 expression^54^. ZEB1 was also confirmed to bind to the TET2 promoter and its knockdown caused an increase in TET2 expression and promoter activity. These results confirm that the downregulation of TET2 by ZEB1 in gliomas is critical in glioma progression. Taken together these data show that ZEB1 may have tumor promoter qualities in glioblastoma and its overexpression leads to poor patient survival as well as increased invasion and metastasis.

However, recent studies have emerged indicating the possibility of a novel tumor suppressive role of ZEB1 in gliomas. One of the first findings were in lower grade gliomas (LGGs) where bioinformatic analysis found increased expression of ZEB1 which positively correlated with a substantial increase in overall survival (OS). Genetic subtype analysis showed that Isocitrate Dehydrogenase 1/2 (IDH1/2)-mutant gliomas, which are gliomas with the best overall prognosis, had a significantly higher expression of ZEB1 and lowered levels of ZEB1 transcriptional target genes^55^. Additionally, these tumors expressed higher levels of miR200c target genes, which as discussed is a key regulator of ZEB1. They further validated these findings and found increased expression of ZEB1 mRNA in IDH1-mutant grades II–III gliomas, and ZEB1 protein expression was more pronounced in these tumors. These findings demonstrate a novel role for ZEB1 in gliomas with the idea that it may have tumor suppressive properties.

**Figure 1:**
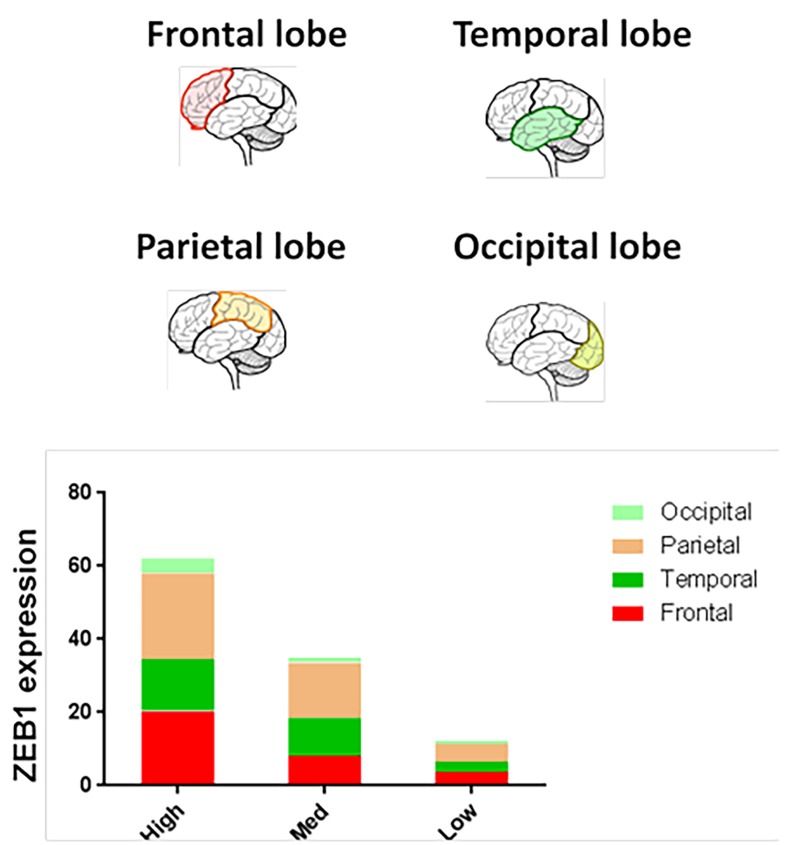
Distribution of ZEB1 expression within identified areas of the brain with gliomas.

A recent short report that wanted to investigate Nesvick’s findings regarding a Chinese population performed a similar analysis using the LGG dataset from the Chinese Glioma Genome Atlas (CGGA)^56^. In this cohort they were able to verify the up regulation of ZEB1 in IDH1/2-mutant LGGs. The downregulation of miR200c was not observed, however, but it is very likely that the regulation of ZEB1 in LGGs is as complex as the cancer itself and may be caused by other mechanisms. Further, no pattern appears to exist regarding where ZEB1 expression is found within gliomas (both and high and low grade) within the brain that can be attributed to tumor suppressor or oncogenic activity (**Figure 1**).

Our own studies have given more confirmation of a tumor suppressive role of ZEB1^57^. Our research studied the role of ZEB1 loss on patient survival in gliomas and in maintaining glioma cancer stem cell (GSC) properties. As cancer stem cells are believed to be at the root for tumor repropagation and poorer patient survival, identifying regulatory genes of these stem cells would be extremely impactful in gliomas. Genomic analysis of over 4000 brain cancers showed that there was a ZEB1 deletion in ~15% (grade II and III) and were significantly higher loss in glioblastomas^57^. These data were verified through such databases as cBioPortal, COSMIC, TCGA and published papers containing information on ZEB1. We further determined that heterozygous deletions in LGGs as well as GBMs were a large reason for ZEB1 loss and that there was frequent loss of heterozygosity (LOH). What was significant with this study was the realization of 3 key components 1) it was necessary to study raw copy number data where heterozygous deletions are more readily identified as opposed to the normal typical analyses of data, thereby explaining the discordance of previous studies that had not revealed decreased expression or loss of ZEB1 in gliomas. 2) A larger dataset <200 patients compared to similar studies of < 20 patients revealed this patient correlation with ZEB1^57^. 3) Lastly, the use of cancer stem cells, which represent a small subpopulation within tumors with seeding and self-renewal capacities, that help contribute to therapy resistance. This often occurs through activation of DNA damage, checkpoint response, and activating/repair cell survival signaling pathways. Many recent studies have shown that activation of EMT can in fact induce the generation and maintenance of CSCs in tumors^58,59^. Cancer stem cells and particularly GSCs have been shown to be genotypically and phenotypically more comparable to gliomas than their conventional glioma cell lines^60-62^ which would be a major factor in some of the discrepancies seen with ZEB1 function. By extension, this would also be consistent in other cancer types where cancer stem cells should be used. The cellular impact of the loss of ZEB1 appeared to be an impairment of stemness. This loss resulted in an increase in the stem cell promoting factor LIF which allows for resistance to differentiation and increased expression of the known stem cell marker CD133 as was shown in experiments involving the knockdown of ZEB1 in GSCs. Further proof of this LIF and ZEB1 interaction was the demonstration of ZEB1 binding regions on the LIF promoter and the repression of LIF by ZEB1. These findings further implicate the tumor suppressive role of ZEB1 in gliomas and indicate that it may perform a significant role in the control of GSCs.

Finally, a study was performed to try and characterize ZEB1 at the level of single cell resolution in human gliomas with respect to clinical and molecular traits. While the study was geared more towards further delineation of conflicting experimental data that suggests that there is a specialized role for ZEB1 at the invasive edge as opposed to a universal effector of oncogenic signaling, their findings were notable when analyzed in light of the previously discussed tumor suppressive properties of ZEB1^63^. Although the LGG sample was too small for statistical analysis they observed the same trend of increased ZEB1 expression in LGGs. It was also shown that in IDH-mutant glioblastomas and glioma cell lines there was uniformity in ZEB1 expression which led them to believe that this ubiquitous and robust expression pattern challenges the canonical concept of ZEB1 as a marker of EMT and tumor progression^63^. One of the most significant findings was that in gross total resections they observed a decrease in the percentage of ZEB1+ cells as they approached the invasive tumor edge challenging previous results which suggest the opposite occurs^49,50^. They also discovered a trend of longer overall survival in tumors with a high ZEB1 labeling index. This would suggest that survival is not negatively correlated with ZEB1 but rather increased expression, if at all a predictor, might serve as a positive predictor of patient survival.

## **5. Conclusion**

With the backdrop of such conflicting data in regard to ZEB1 in gliomas, it is necessary that more studies be done to further elucidate the roles that ZEB1 truly plays. Studies that deal with larger patient cohorts would be more informative in determining the role of ZEB1 at least in gliomas. Through the use of large patient cohorts we have recently reported ZEB1 to be a predictive and prognostic marker in diffuse gliomas^64^. Given the dual nature of ZEB1 with respect to its ability to act as a transcriptional repressor or activator, one needs to determine if the coactivators or corepressors that are associated with ZEB1 are in fact functional in glioblastoma and are not subject to mutational events or other dysregulation that may affect ZEB1. This can further be extended as new information is obtained regarding the interplay between ZEB1 and mirco-RNAs, long coding RNAs and potential epigenetic mechanisms that may influence ZEB1 activity. Similarly, questions abound regarding ZEB1 activity that still need to be considered, for example-Is it possible that ZEB1 is able to alter its function based on environmental pressures? Is there a subpopulation basis to the duality that is observed in regard to transcriptional activation or repression? In a heterogenous cancer such as glioblastoma it is possible that there are areas of high and low to null ZEB1 expression. While the tumor suppressive role of ZEB1 remains controversial and is still in its infancy, ZEB1 research as it pertains to gliomas and other cancers is undeniably complex and still has much that remains to be unveiled.

## **KEY POINTS**

**◊** **ZEB1 is a critical component of the EMT process in normal and cancer development**

**◊** **Evidence is building for ZEB1 being a tumor suppressor**

**◊** **More research is needed to unravel ZEB1 complexity in cancer**
